# P042: Severe influenza infections requiring intensive care during winter 2012/2013

**DOI:** 10.1186/2047-2994-2-S1-P42

**Published:** 2013-06-20

**Authors:** C Landelle, A Iten, L Kaiser, Y Thomas, E Genevois, D Joubert, J-C Richard, S Harbarth, D Pittet, L Brochard

**Affiliations:** 1 Infection Control Program, University of Geneva Hospitals, Geneva, Switzerland; 2 Laboratory of Virology, University of Geneva Hospitals, Geneva, Switzerland; 3 Intensive Care Unit, University of Geneva Hospitals, Geneva, Switzerland

## Introduction

Seasonal influenza (SI) is usually a benign self-limited disease. However, it can present a serious health threat.

## Objectives

We describe influenza cases admitted to a 34-bed adult intensive care unit (ICU) in a Swiss university hospital during winter season 2012/2013.

## Methods

From 28/11/2012 to 11/03/2013, among 268 hospitalized cases of SI confirmed through nasopharyngeal samples using real-time PCR, 34 were admitted to the ICU. Droplet precautions were applied to all patients (pts) at ICU admission without single room isolation.

## Results

23 pts (68%) were positive for influenza A and 11 (32%) for influenza B. 18 pts (53%) were male; median age was 63 years (interquartiles range [IQR]: 47-74]); 29 pts (85%) presented a least one co-morbidity and 19 (56%) had risk factors for SI: chronic respiratory disease (n=12), diabetes (n=7), obesity (n=4), chronic cardiac disease (n=4), immunosuppression (n=3) and pregnancy (n=1). Only 5 pts had received influenza vaccination. 29 cases were community-acquired (85%) while 5 were hospital-acquired (15%) (symptoms occurring >3 days [d] after admission). No ICU-acquired influenza was detected. 9 pts (26%) had a documented co-infection with: *S. pneumonia* (n=5), *S. pyogenes* (n=2), methicillin-sensitive *S. aureus* (n=1) and Coronavirus (n=1). 27 pts (79%) were treated with oseltamivir, with a median delay after onset of symptoms of 5 d (IQR: 3-7). 27 pts (79%) needed ventilatory support: invasive ventilation (n=17 [50%] for a median duration of 6 d [IQR: 2-13]) or non-invasive ventilation (n=15 [44%] for a median duration of 2 d [IQR: 2-3]). The median length of ICU and hospital stay were 5 (IQR: 3-9) and 11 d (IQR: 7-23), respectively. 5 pts (19%) died of influenza-related complications, including 1 case of nosocomial superinfection.

## Conclusion

Winter season 2012/2013 was characterized by a massive burden of cases in the ICU, with no co-morbidity for 15% of the patients. Despite increased workload, use of face mask ventilation and no isolated rooms, it is remarkable that no clinical case of transmission was recognized or microbiologically documented inside the ICU during this period.

## Disclosure of interest

None declared

